# Two Rare Surgical Cases

**Published:** 1857-07

**Authors:** Wm. M. Boling

**Affiliations:** Montgomery, Alabama


ART. VII.—*Two rare Surgical Cases.* By WM. M. BOLING, M.D., of Montgomery, Alabama.

1. *Large and repeated Hemorrhages from the socket of a tooth, dependent upon Necrosis of the Jaw-bone.*—On the 22d of May, 1851, I visited a man, about 28 years old, living some fifteen miles from Montgomery, and received from Dr. Wilson, his attending physician, the following history of his case. About the 1st of March, preceding my visit, the patient had an attack of erysipelas of the right side of the face. Very great swelling of the part took place, into which, in the course of treatment, an incision was made just below the eye, with the effect of discharging some matter. On the eighteenth of the month, he had a slight hemorrhage from the opening made with the lancet, from the right nostril, and also from a small abscess that had formed near the root of the second molar tooth. On the 21st, he had another slight hemorrhage from the same parts, a more considerable one on the 24th, and also on the 27th. About the 1st of April, the parts continuing very much swollen and extremely painful, the first molar tooth and a root of the adjoining bicuspid, were extracted, from the sockets of which a small quantity of offensive matter escaped. On the 13th of April, he had a profuse hemorrhage, estimated at about two quarts, the loss of which produced syncope, principally from the socket of the extracted tooth; on the 25th, the same again, resulting in great prostration and syncope, and on the 21st of May it was again repeated, to the same extent, and with the same result. The blood was apparently arterial. In each instance the discharge ceased seemingly in consequence of the prostration, and was renewed, apparently, whenever, from improvement in the quality and restoration of the quantity of the blood, the system had, to some extent, recuperated.

When I saw the patient on the 22d, the hemorrhage had ceased, and a reaction, more irritative than febrile, had taken place. There was some heat of the surface more than natural; the pulse was 100, quick and jerking; the lips were white and bloodless; there was a tendency to œdema, and the prostration was extreme; though, prior to the attack, the patient was quite a strong and robust man.

The side of the face, more especially over the maxillary sinus, was swollen and prominent, and within the mouth, between the cheek and the alveolar margin, there was a bony projection, as if the lower anterior part of the wall of the antrum was pressed downward and forward by a body filling the cavity.

Though the patient seemed but little able to survive the loss of blood from many more such attacks, and as it was not probable, considering his present prostrate condition, that another hemorrhage would occur for some time, it was decided, in view of the uncertainty as to the real character of the case, not to interfere actively for the present. In consideration of the extreme danger to which the patient was reduced by the recurring hemorrhages, and more especially as it seemed not improbable that these proceeded from a vascular growth filling the antrum, the propriety of applying a ligature to the carotid was taken under consideration; and it was decided to be prepared at least for such a measure, in any operative course adopted, should emergency require it.

On the 30th of May, as it was apprehended that a return of the hemorrhage might soon take place, I visited the patient again. The parts seemed in much the same condition. A probe inserted into the opening on the cheek made with the lancet, readily passed under the integuments as far as the outer or inner canthus, and an inch or more backward along the floor of the orbit. On pressing the finger into the orifice from which the tooth had been extracted, and from which the great discharges of blood had proceeded, a projecting point or margin of apparently denuded bone could be felt. A probe could be passed readily into the antrum. I introduced a bistoury into the opening, and divided the substance of the gum for about three-eighths of an inch, in the two directions, backwards and forwards, and then, with a pair of forceps introduced through the opening, succeeded in removing some five or six pieces of exfoliated bone, differing in size from that of the little finger nail, to nearly that of a quarter of a dollar; seemingly the internal bony parietes of the antrum. After the operation, the end of the forefinger could be readily passed into this cavity. No hemorrhage of any consequence occurred during the operation.

I have not seen the patient since the slight operation by which he was apparently relieved from such extreme danger, but have heard from him within the past year. There was no return of hemorrhage. The swelling of the face subsided in a short time, and the recovery was prompt and permanent.

2. *Perforation of the Popliteal Artery and Vein by a Spiculated Exostosis of the Femur; Large Extravasation of Blood mixed with Pus. Amputation. Recovery.* On the 1st of August 1849, I visited a young man, living in an adjoining county and under the professional care of Doctors Smith, Hillhouse, and Jones. He was about sixteen years old, and before the attack for which I visited him, was well grown, strong, athletic, and of very active habits. Some five or six months before the date of my visit, he felt pain about the knee and popliteal region, slight at first, but rapidly increasing in severity. At first, the swelling was slight, but, after some weeks, it increased more rapidly, more especially in, and apparently starting from, the popliteal space. For the last six weeks the patient has been confined all the time to bed. His suffering is great; he is very much emaciated and his pulse is quick (120); and small. At no time has the swelling been circumscribed, presenting the defined limits of a tumor, but rather diffused. It involves the entire circumference of the leg, which at the largest part, just above the knee, measures 23 inches—more than twice as much as the sound leg at the corresponding point. The enlargement extends from just below the knee towards Poupart's ligament, 14 inches. It has rather a doughy feel, but, at times, is more elastic. There is no pulsation, but an obscure, peculiar, muffled, *bruit de soufflet*, more distinct at the most prominent point than at the margin, over the femoral artery. There is no pulsation in the anterior tibial artery where it passes over the dorsum of the foot. A peculiarity about the tumor is that, at times it suddenly enlarges, increasing greatly in size in a few minutes, becoming tense and much more painful. This increase in size, though coming on suddenly, very gradually subsides, to be renewed at irregular and uncertain periods. On inserting an exploring needle, a mixture of pus and grumous blood was discovered, and, on enlarging the opening with a lancet (we had previously decided on amputation), the same matter continued to escape slowly for about a minute; then a small coagulum was thrown out with force, followed by a stream, slightly *per saltum*, of mixed arterial and venous blood. Though the orifice was promptly closed, the effect of even this slight loss of blood was manifest upon the pulse.

We were at a loss in regard to the diagnosis. Some of the characters of aneurism were present; but it was surely not an ordinary aneurism. Whatever the real nature of the case, however, the removal of the limb seemed to offer the only chance to the patient of recovery. Already reduced as he was to the verge of the grave, it seemed scarcely probable, even supposing the case to be one of ordinary aneurism, but of unusual size and attended with unusual local suffering, that merely tying the artery would cause an abatement of the irritation, sufficient and early enough to preserve life. Accordingly, after having the patient placed under the influence of chloroform, I amputated the limb near the hip joint. Though the loss of blood was very slight, the patient, already so much prostrated, sunk into a state of extreme collapse, through which he was sustained, and from which he was recovered in the course of the night, by the persevering administration of stimulants. It was the opinion of the gentlemen named, who were present, and I thought myself, that the patient could not have survived the operation but for the sustaining influence of the chloroform and its prevention of nervous shock. He was not aware till the stump was dressed, that the leg had been amputated.

The femur was found rough, and very slightly enlarged just above its lower articulating surface; and extending backwards from between the condyles, was a projecting conical point of bone about half an inch in length, by which both the popliteal artery and vein were perforated. There was, of course, no regular aneurismal sac, but the blood had been forced, as it were, in every direction and into every possible point, separating the muscles and filling the interstices between them. It was estimated that the coagula, could all have been saved, would have weighed ten pounds. After the dissection, a quantity weighing seven pounds was collected. The patient's recovery was speedy and uninterrupted.

